# Rehabilitación de la baja visión: un asunto incipiente* **

**DOI:** 10.15649/cuidarte.2665

**Published:** 2023-09-06

**Authors:** María del Pilar Oviedo-Cáceres, Karen Natalia Arias-Pineda, Diana Cristina Palencia-Flórez

**Affiliations:** 1 . Universidad Santo Tomás. Bucaramanga. Colombia. Email: maría.oviedo@ustabuca.edu.co Universidad Santo Tomás Universidad Santo Tomás Bucaramanga Colombia maría.oviedo@ustabuca.edu.co; 2 . Universidad Santo Tomás. Bucaramanga. Colombia. E-mail: karen.arias01@ustabuca.edu.co Universidad Santo Tomás Universidad Santo Tomás Bucaramanga Colombia karen.arias01@ustabuca.edu.co; 3 . Universidad CES. Medellin. Colombia. Email: palencia.diana@uces.edu.co Universidad CES Universidad CES Medellin Colombia

**Keywords:** Baja Visión, Servicios de Salud, Rehabilitación, Acceso a Servicios, Vision, Low, Health Services, Rehabilitation, Access of Health Services, Baixa Visáo, Servigos de Saúde, Reabilitagáo, Acesso a Servigos de Saúde

## Abstract

**Introducción:**

Las personas con baja visión requieren de un proceso de rehabilitación de la visión que les permita optimizar su resto visual, mitigando así el impacto de la discapacidad.

**Objetivo:**

Describir las condiciones del acceso a la rehabilitación de la visión en Bucaramanga y su Área Metropolitana.

**Materiales y Métodos:**

Se realizó un estudio de caso colectivo tomando elementos de la fenomenología. Se asumió la postura teórica de Andersen y colaboradores. Se realizaron entrevistas semi-estructuradas a 11 pacientes y profesionales involucrados en la atención y rehabilitación, residentes en municipios del área metropolitana de Bucaramanga. El análisis se hizo en tres momentos: descubrimiento, codificación e interpretación de los datos.

**Resultados:**

Dos categorías emergen del estudio: 1. Rehabilitación de la visión: Un asunto incipiente. 2. Experiencia de las personas con baja visión frente a los procesos de atención para el manejo de la baja visión, con sus subcategorías: Fallas en la identificación y orientación frente al manejo de la baja visión y Dificultades para asistir a las atenciones clínicas y acceso a dispositivos.

**Discusión:**

Describir las condiciones de acceso a los servicios de rehabilitación puede contribuir a generar estrategias de intervención que permitan abordar las barreras identificadas.

**Conclusiones:**

Las consecuencias de la baja visión pueden ser atenuadas al acceder a procesos de rehabilitación; sin embargo, en los cuatro municipios participantes las personas experimentan múltiples barreras para lograr su rehabilitación, lo que evidencia la necesidad de establecer mecanismos que permitan el ejercicio del derecho a la salud de las personas con discapacidad visual.

## Abstract (en)

**Introduction:**

People with low vision need a vision rehabilitation process that allows them to optimize their remaining vision and thus mitigate the impact of the disability.

**Objective:**

To describe the conditions for access to vision rehabilitation in Bucaramanga and its metropolitan area.

**Materials and Methods:**

A collective case study was conducted by taking elements from phenomenology. The theoretical position of Andersen and collaborators was assumed. Semi-structured interviews were conducted with 11 patients and professionals involved in care and rehabilitation residing in municipalities in the metropolitan area of Bucaramanga. The analysis was done in three stages: discovery, coding, and interpretation of the data.

**Results:**

Two categories emerge from the study: 1) Vision rehabilitation: An incipient issue, and 2) People with low vision experience in low vision management care processes; the latter with the following subcategories: Failures in the identification and orientation to low vision management and difficulties in attending clinical care and accessing devices.

**Discussion:**

Describing the conditions for accessing rehabilitation services can contribute to designing intervention strategies to address the identified barriers.

**Conclusions:**

The consequences of low vision can be mitigated by accessing rehabilitation processes; however, in the four participating municipalities, people face multiple barriers to rehabilitation. This situation evidences the need to establish mechanisms that allow people with visual impairments to exercise their right to health.

## Resumo

**Introdujo:**

As pessoas com baixa visao necessitam de um processo de reabilitagáo visual que lhes permita otimizar o descanso visual, mitigando assim o impacto da deficiencia.

**Objetivo:**

Descrever as condigóes de acesso a reabilitagáo visual em Bucaramanga e sua Área Metropolitana.

**Materiais e Métodos:**

Realizou-se um estudo de caso coletivo a partir de elementos da fenomenologia. foi assumido posigáo teórica de Andersen e colaboradores. Foram realizadas entrevistas semiestruturadas com 11 pacientes e profissionais envolvidos no cuidado e reabilitagáo, residentes em municípios da área metropolitana de Bucaramanga. A análise foi feita em tres momentos: descoberta, codificagáo e interpretagáo dos dados.

**Resultados:**

Duas categorias emergem do estudo: 1. Reabilitagáo da visáo: uma questáo emergente. 2. Vivencia das pessoas com baixa visáo quanto aos processos de cuidado para o manejo da visáo subnormal, com suas subcategorias: Falhas na identificagáo e orientagáo quanto ao manejo da visáo subnormal e Dificuldades no atendimento clínico e acesso aos dispositivos.

**Discussao:**

Descrever as condigóes de acesso aos servigos de reabilitagáo pode contribuir para gerar estratégias de intervengáo que permitam enfrentar as barreiras identificadas.

**Condusdes:**

As consequencias da baixa visáo podem ser mitigadas com acesso a processos de reabilitagáo; no entanto, nos quatro municípios participantes, as pessoas enfrentam múltiplas barreiras para alcangar sua reabilitagáo, o que mostra a necessidade de estabelecer mecanismos que permitam as pessoas com deficiencia visual exercer o direito a saúde.

## Introducción

La baja visión es una deficiencia visual moderada o grave, que genera limitaciones para el desarrollo de actividades cotidianas, producto de una patología de base^1^. Para el año 2015, se reportó que en el mundo existen 36 millones de personas ciegas y 216 millones con baja visión^2^^,^^3^. Datos preliminares del Censo del 2018 muestran que la discapacidad visual es la segunda de mayor prevalencia en el país^4^. Para el caso específico de las ciudades donde se llevó a cabo este proyecto, se reportaron las siguientes cifras de discapacidad visual: Bucaramanga 2.434 Floridablanca 1.698, Girón 196 y Piedecuesta 440^5^.

Teniendo en cuenta la clasificación de la Organización Mundial de la Salud dadas en la CIE10R y CIE- 11, se define la baja visión en términos de la clasificación de rangos de agudeza visual (AV); en este sentido se entiende como una AV mejor corregida peor que 20/60 pero igual o mejor que 20/400 en el mejor ojo o pérdida de campo visual correspondiente a menos de 20° en el mejor ojo con la mejor corrección posible^6^. Los servicios de atención para la rehabilitación de la visión buscan optimizar la funcionalidad de la persona a través de maximizar el uso de la visión residual y ofrecer adaptaciones prácticas para afrontar las consecuencias de la deficiencia visual en el ámbito social, psicológico, emocional y económico^6^.

La rehabilitación de la visión puede ser definida como la combinación de intervenciones sanitarias, educativas y sociales cuyo objetivo final es reducir el impacto negativo de la baja visión al maximizar la función visual^7^. Pese a lo anterior, sólo el 5% de la población global tiene acceso a servicios de rehabilitación y solo 1 de cada 10 personas que necesitan productos de apoyo tiene acceso; se ha reportado que 200 millones de personas con esta discapacidad visual, carecen de acceso a dispositivos para mejorar la visión^8^^,^^9^.

En Colombia, solo se rastrea un estudio previo que busca indagar asuntos relacionados con el acceso a servicios de salud de baja visión^10^^,^^11^, el cual no consideró la perspectiva de los pacientes, razón por la que este estudio buscó describir las condiciones de acceso a la rehabilitación de la visión en Bucaramanga y su Área Metropolitana como mecanismo inicial para identificar fortalezas y debilidades que puedan ser abordadas con el fin de contribuir a la mejora de la atención en salud de este grupo poblacional.

## Materiales y Métodos

La investigación se enmarca en el paradigma cualitativo, se realizó un estudio de caso colectivo^12^^,^^13^ entendido como un sistema integrado, compuesto por estudios individuales, es decir por unidades de análisis, que comparten características comunes y que, si bien se estudiaron en su singularidad, luego, a partir de ellos, se obtuvo una mejor comprensión de las experiencias de las personas con baja visión y su búsqueda para el acceso a servicios de rehabilitación. Para el abordaje del estudio de caso se tomaron elementos de la fenomenología dado que permite explorar el fenómeno en su escenario específico y asumir como eje central la experiencia vivida por las personas en relación con el objeto de estudio. Se asumió la postura teórica de Andersen y Colaboradores^14^^,^^15^, quienes mencionan que para el estudio del acceso a los servicios de salud se tienen en cuenta dos dimensiones: el acceso potencial y el acceso real.

Se realizó un muestreo intencional y por bola de nieve utilizando referencias de informantes clave y participantes^16^. La selección de los participantes utilizó el criterio de entrevistado especial, definido como aquellos que se ubican en una posición única en la comunidad, para ser considerados en la investigación, las personas debían ser mayores de edad, tener diagnóstico de baja visión, vivir en Bucaramanga, Girón, Piedecuesta o Floridablanca o ser profesionales de la salud dedicados a la rehabilitación de personas con esta condición. En la Tabla1 se describen los participantes del estudio.

**Tabla 1:** Descripción de participantes del estudio

Perfil	Número de participantes	Municipio
Persona con baja visión	2	Bucaramanga
	2	Girón
	2	Floridablanca
	2	Piedecuesta
Prestadores de servicio de salud	2	Floridablanca
	1	Bucaramanga
Total de personas participantes	11	

Se acudió al uso de técnicas de recolección de información que permitieran la conversación y el intercambio de experiencias entre los sujetos y las investigadoras, en este sentido se realizaron entrevistas semi-estructuradas las cuales permitieron captar vivencias, opiniones y concepciones sobre el acceso a los servicios de rehabilitación, así como los elementos críticos, que, de acuerdo con la trayectoria de cada persona, pueden estar afectando significativamente el acceso real a los servicios en las ciudades de estudio. Las preguntas planteadas a los profesionales involucrados en la atención y rehabilitación de personas con baja visión estuvieron orientadas hacia: ¿Cuál es el proceso que debe seguir una persona para acceder a los servicios? ¿Cuáles son las mayores dificultades que se enfrentan en la rehabilitación? Por su parte las preguntas para las personas con baja visión estuvieron enfocadas hacia: ¿Cómo fue su proceso para llegar a una atención de rehabilitación de la visión? ¿Cuáles han sido las barreras que ha encontrado en el proceso?

Teniendo en cuenta las condiciones sanitarias por el brote de la COVID-19 y en algunos casos por las ocupaciones personales y profesionales que no hicieron posible el encuentro presencial, se realizaron entrevistas semiestructuradas telefónicas, las cuales fueron grabadas en medio digital, mediante el uso de una aplicación para tal fin. Una vez realizadas se guardaron e identificaron teniendo en cuenta la ciudad y el rol del entrevistado asignándose un número a cada una; posteriormente se transcribieron en su totalidad. Para el análisis de la información, se utilizó el análisis cualitativo propuesto por Taylor, siguiendo el momento de descubrimiento, codificación y relativización o interpretación de los datos^17^.

Se implementó la estrategia de triangulación de investigadoras, pues cada una codificó de forma independiente las transcripciones; los códigos y categorías resultantes se refinaron a través de reuniones de equipo en curso para participar en la reflexión colectiva, ampliando las posibilidades de codificación. Las transcripciones de las entrevistas se encuentran en Mendeley Data^18^. Para apoyar el análisis se utilizó el software atlas.ti (v. 6.2). Todas las personas invitadas aceptaron participar y luego se solicitó su consentimiento informado verbal. En todo momento se garantizó el manejo ético, anónimo y confidencial de la información y del nombre de los participantes. Esta investigación contó con el aval del comité de ética de la Universidad Santo Tomás de Bucaramanga.

## Resultados

### Rehabilitación de la visión: Un asunto incipiente

El panorama frente a los procesos de rehabilitación de la visión en los cuatro municipios participantes de la investigación es incipiente y con muchas falencias, pues en el mapeo de actores solamente se identificaron dos instituciones de salud con servicios especializados para dar atención a las personas con baja visión: La primera de tipo privado ubicada en Bucaramanga, que presta servicios bajo la modalidad particular y a usuarios de una aseguradora en el marco del sistema de salud y la segunda, ubicada en Floridablanca, vinculada a una institución universitaria que presta servicios bajo la modalidad docencia servicio. Estos elementos de entrada son críticos si se tiene en cuenta que los recursos humanos y de infraestructura son fundamentales para el acceso a los servicios de salud y de rehabilitación, dada la alta prevalencia de discapacidad visual en Bucaramanga y su área Metropolitana^5^. La Tabla 2 muestra las características de las instituciones:

**Tabla 2:** Características de instituciones de salud con servicios de baja visión

Tipo de institución	Número de profesionales vinculados a la atención	Horas de dedicación a la semana	Servicios prestados	Tipos de pacientes atendidos	Proceso de rehabilitación
Privada/ Docencia servicio	2	4	Evaluación diagnóstica en baja visión	Particulares	No
Privada	1	Dependiendo de la demanda al servicio	Prescripción de dispositivos ópticos	Atención en el marco del sistema de salud y particulares	No

El proceso de atención en salud de las personas con baja visión requiere de un abordaje integral en el que se incluyen diferentes intervenciones y profesionales que, de manera conjunta, contribuyen en aminorar la experiencia de la discapacidad de las personas con baja visión; sin embargo, como se muestra en la Tabla 2, ninguna de las instituciones realiza un proceso de rehabilitación funcional visual multidisciplinario, pues solamente se hace prescripción y entrenamiento en los dispositivos ópticos.

*“Es una consulta de baja visión, paremos ahí. Yo llego hasta el punto de formular y prescribir la ayuda. Realmente rehabilitación no hacemos..." [EProfesional #1].*

En este mismo sentido el profesional de la otra institución manifiesta

*“Se hace un proceso para medir el residuo visual, tener un conocimiento más profundo y empezar a pensar en las posibles opciones. Luego es hacer propuestas en cuanto a ayudas ópticas y no ópticas de cómo podemos ayudarle a la persona a que pueda cumplir con esos objetivos, pero una rehabilitación completa como se debiera, no la hacemos" [EProfesional #2]*

### Experiencia de las personas con baja visión frente a los procesos de atención para el manejo de la baja visión

#### Fallas en la identificación y orientación frente al manejo de la baja visión

Las personas entrevistadas manifiestan dificultades para el proceso de búsqueda de servicios de rehabilitación para la condición de baja visión, pues en primera medida no obtienen una adecuada orientación frente a las posibilidades de atención.

*“No sabíamos qué proceso seguir después de que uno pierde la visión, porque no recibí ninguna asesoría, ningún especialista me orientó sobre el tema de los ojos” [EPBV #1]*

Estas dificultades iniciales con relación a la falta de orientación dada por los profesionales de la salud fueron vividas por todas las personas participantes del estudio, aspecto que pone en evidencia la necesidad de abordar esta temática desde la formación del recurso humano en salud, pues éstos tienen la responsabilidad ética de brindar la información necesaria al paciente y su familia para hacer frente a las transformaciones cotidianas que implica la discapacidad visual.

*“A mí no me han hablado de la discapacidad visual, lo único que me han dicho es que tenga paciencia que ya no tengo solución” [EPBV #4].*

En este sentido, emerge la percepción que tienen algunos de los profesionales de la salud frente al concepto de la baja visión, pues para algunos de ellos esto no se configura como una condición de discapacidad visual, aspecto que de entrada limita las posibilidades de las personas, por ejemplo, para el acceso de servicios de rehabilitación o de programas sociales para su inclusión social.

*“Yo no les digo que tienen baja visión o discapacidad porque para mí ellos no tienen discapacidad. Yo les hablo de la patología y de la forma como la deben tratar, pero no me gusta usar esa palabra” [EProfesional #3].*

*“Nosotros no hablamos de discapacidad, hablamos de glaucoma, degeneración macular, preferimos evitar ese término” [EProfesional #2].*

En la medida en que las personas no conozcan su condición visual, todo el panorama que implica su situación de salud se limita principalmente lo correspondiente a los procesos para su inclusión social.

Las personas en su mayoría llegan a los servicios de baja visión mediante el mecanismo de voz a voz, mediante el reconocimiento de pares que también tienen su condición y no por derivación directa de profesionales de la salud, aspectos que permiten dar cuenta de las falencias en términos de cultura de referencia o de identificación de este tipo de condición en la práctica clínica.

*“Al principio lo más complejo es conocer las opciones, si de casualidad mi hermana no conoce a alguien con esta condición, no sabemos que existía un proceso de rehabilitación.” [EPBV #2].*

En este mismo sentido otro participante manifiesta

*“Una tía me dijo que había otro familiar que estaba yendo a la escuela para ciegos que yo por qué no iba a averiguar”. [EPBV #5]*

Estas dificultades son reconocidas por las personas entrevistadas como una barrera importante para su proceso de atención, pues en la medida que los profesionales no dan la información y la orientación, las probabilidades de lograr una atención y rehabilitación son mínimas.

*“Los especialistas en la parte de visión son los primeros que deberían asesorarnos, pero es difícil si a uno no le dicen que hay procesos más avanzados para uno rehabilitarse”. [EPBV #6].*

Un aspecto que se desencadena como producto de la barrera mencionada anteriormente tiene que ver con los largos tiempos que esperan las personas para lograr identificar un servicio de atención y el impacto que esto puede tener para la continuación de sus actividades laborales, educativas, de ocio y en fin último su inclusión social

*“Uno puede perder muchos años, gracias a Dios yo no perdí tantos, pero he escuchado personas en las rehabilitaciones que tienen 40 o 50 años y hasta ahora están empezando”. [EPBV #3].*

En esta misma ruta otra persona participante manifiesta

*“Ya cuando llevaba como dos años de haber perdido la visión, una doctora en los controles me dijo que existían programas de rehabilitación.” [EPBV #4]*

Pese a lo anterior, se identifica una experiencia positiva frente a la derivación al servicio, pues una de las personas entrevistadas obtuvo asesoría por parte de los profesionales tratantes y de esta manera pudo evitar las barreras experimentadas por las demás personas participantes.

*“Yo fui donde un retinólogo y me dijo ya acostúmbrese a ver lo que pueda, pero vaya al programa de baja visión, la doctora me orientó y me mando aquí”. [EPBV #6].*

Este tipo de experiencias da cuenta de la importancia y del impacto que tiene la adecuada orientación frente a las posibilidades de manejo de la discapacidad, poniendo de relevancia el rol que tienen los profesionales de oftalmología y optometría en esta situación dado que son los encargados del cuidado primario de la salud visual y ocular y de su rehabilitación.

#### Dificultades para asistir a las atenciones clínicas y acceso a dispositivos

Una vez se logra la obtención de la identificación de instituciones de salud, emerge una nueva barrera relacionada con los gastos de bolsillo que asumen las personas y sus familias para el desplazamiento a los centros de atención.

*“Dejé de ir porque ya me quedaba muy difícil ir desde Piedecuesta hasta Bucaramanga porque nos fuimos a vivir allá”. [EPBV #4]*

A este panorama se suma el hecho de que algunos de ellos deben acudir con un acompañante pues en algunos casos tienen restricción para su movilidad segura en espacios públicos y esto genera por tanto mayores dificultades y gastos de bolsillo.

*“La venida es difícil porque no tengo quien me acompañe, he perdido dos citas, porque no tengo quien me acompañe y me da pena pedirle a alguien que uno no conozca.” [EPBV #5]*

Sumado a lo anterior se incluyen los gastos para el pago de las consultas, pues como se mencionó anteriormente las instituciones de salud son de carácter privado y por tanto todos los servicios deben ser pagados particularmente. Desde el Sistema de Salud Colombiano actualmente solo se cubre la atención diagnóstica en baja visión y el acceso a los dispositivos se hace vía tutela o Mipres; sin embargo, una de las instituciones actúa por fuera del sistema razón por la cual no puede gestionar estos trámites y en este sentido sus pacientes deben acceder a los servicios vía pago directo. Ante esto, una persona que acude a la institución de Floridablanca menciona

*“Este tratamiento no lo cubre y son veinte mil pesos cada vez que vengo” [EPBV #3].*

De la misma manera otra persona manifiesta

*“Todo eso fue particular lo costeó mi familia” [EPBV #6].*

Finalmente, una de las personas entrevistadas que acude al centro de Bucaramanga comenta

*“Los gastos para la rehabilitación me apoyan mis hijas, el seguro no cubre nada” [EPBV #5]*

La intervención que se realiza en las dos instituciones está encaminada al diagnóstico de la baja visión y a la prescripción y orientación frente al uso de los dispositivos de apoyo, tales como lupas, telescopios, entre otros. En este punto aparecen barreras para los pacientes, pues por una parte estos elementos no están incluidos en el sistema de salud y por otro los costos para su adquisición son elevados.

*“Difícil es trabajar sin herramientas, porque no solamente tengo las barreras de la Universidad, que no puedo hacer sino hasta aquí, sino que también tengo las barreras de que si le doy una ayuda el sistema no se la da”. [EProfesional #2]*

En este sentido desde la perspectiva de las personas con baja visión, este asunto también se identifica como una falencia

*“Acceder a las ayudas es poco fácil para las personas porque por lo general está asociado a una condición de bajos recursos económicos y sabemos que son costosas para una persona que no trabaja y si a eso se le suma la situación económica familiar y toda la inversión que se supone tener un hijo con discapacidad” [EPBV #1]*

Estas barreras económicas son percibidas por los profesionales encargados de la atención de baja visión.

*“Muchos de los pacientes que atendemos vienen de lejos y tienen dificultades para pagar las citas y mucho más las ayudas ópticas, entonces es muy difícil” [EProfesional #3]*

Para los pacientes que son atendidos por la institución de la ciudad de Bucaramanga y que acceden vía aseguradora, se abre un camino para obtenerlos mediante mecanismos como tutela o Mipres;

*“A principios de este año iba a hacer los trámites para ver si podía acceder a ellos y el doctor me dijo que hiciera el trámite a ver si por el lado de la tutela se podía, entonces estamos en eso, pero no es fácil” [EPBV #3]*

Lo anterior implica someter la rehabilitación a un proceso de trámites legales que dilatan la posibilidad de acceder de manera oportuna y ágil a mecanismos que les permitan mejorar sus condiciones de vida. Sin embargo, no todos logran un resultado positivo, pues no se les autoriza la totalidad de los dispositivos

*“No pude acceder a las lupas por costos, pues pensé en comprarlos de manera particular, le pasé una tutela a la EPS pero no me aprobaron” [EPBV #2]*

Desde la perspectiva de las personas entrevistadas, los servicios de baja visión deberían mejorarse e incluirse de una manera más clara en las atenciones que se realizan en las EPS.

*“El gobierno debería hacer unos planes en donde uno pueda rehabilitarse y ser más útil para la sociedad, todo nos ha tocado muy rebuscado”. [EPBV #1]*

## Discusión

La baja visión es una condición cada vez más frecuente lo cual se explica entre otras, por el crecimiento poblacional y el envejecimiento^19^, aspectos que ponen de manifiesto la necesidad abordar integralmente las necesidades de rehabilitación de este colectivo.

Las consecuencias de la baja visión pueden ser atenuadas al acceder a procesos de rehabilitación, que buscan optimizar el remanente visual funcional, una premisa concordante con lo descrito por la OMS y lo mencionado en la ley Estatutaria 1618 de 2013 en Colombia, que indica que el fin de las intervenciones en personas con discapacidad consiste en dotarlas de técnicas, estrategias y recursos que contribuyan a la realización de actividades cotidianas en cualquier entorno con el fin de contribuir a una integración social acorde con sus expectativas; sin embargo y teniendo en cuenta los hallazgos emergentes de la presente investigación podemos identificar que en los cuatro municipios participantes las personas experimentan múltiples barreras para lograr su rehabilitación, es importante mencionar que ninguna de las personas entrevistadas ha realizado un proceso completo en este sentido y por el contrario ha recibido intervenciones puntuales y muy acotadas al asunto visual pero no a las implicaciones arriba mencionadas de la discapacidad visual.

Tal y como se ha mencionado en otros estudios, la rehabilitación de la baja visión no se debe limitar simplemente a recomendar ayudas como gafas telescópicas o lupas^20^. Más importante es la formación en el uso de estos dispositivos y el proceso de rehabilitación interdisciplinario^21^. Desde el consenso internacional de Roma se ha venido hablando sobre la necesidad de trascender el enfoque instrumental y puntual de la prescripción de dispositivos ópticos y hablar de la rehabilitación como un continum que acompaña a la persona y su familia para hacer frente a los retos que implica la discapacidad visual^22^.

Las complejas barreras mostradas en este estudio son coherentes con lo reportado en el contexto internacional, pues solo entre el 3% y el 15% de los pacientes logra acceder a un proceso de rehabilitación^23^. Las principales causas de estas barreras están asociadas con la falta de conocimiento de profesionales sobre criterios de derivación, dispositivos de rehabilitación y los beneficios que traen para sus pacientes. Además, falta de conciencia de profesionales sobre los servicios de rehabilitación. Algunos estudios muestran que los profesionales de la salud consideran que no eran buenos candidatos para derivar aquellas personas residentes de zonas rurales, analfabetas, mujeres de bajo nivel socioeconómico, personas con discapacidad física, menores de cinco años y quienes psicológicamente no estén preparados^24^^,^^25^. De igual manera las barreras experimentadas por las personas participantes de este estudio son similares a las mencionadas en estudios previos en donde se muestra que las personas no son derivadas a los servicios, no tienen conocimiento sobre opciones de atención, dificultades de transporte y falencias en la comunicación con el profesional^26^^,^^27^.

Un aspecto crítico de este estudio tiene que ver con los temores asociados al uso de la palabra discapacidad o baja visión por parte de los profesionales de la salud, lo cual hace que las personas no tengan toda la información necesaria sobre su situación de salud y de esta manera inmediatamente se les está restringiendo sus posibilidades de atención y rehabilitación. Es importante trabajar en la formación de estos profesionales de la salud para abordar de manera integral la discapacidad visual desde un enfoque de derechos humanos, dándoles herramientas para la comunicación de este tipo de situaciones en el contexto de una atención clínica.

## Conclusiones

Existe una limitada oferta de instituciones y profesionales dedicados a la atención en baja visión, así mismo no se realizan procesos de rehabilitación de la visión, pues solamente se hacen intervenciones clínicas diagnosticas en baja visión y prescripción de dispositivos. Es importante hacer una labor de abogacía en los municipios, que permita mostrar la importancia de mejorar el acceso a los servicios de rehabilitación para personas con discapacidad visual. De la misma manera, es prioritario fortalecer la formación del recurso humano en salud visual para hacer frente a las necesidades de rehabilitación visual.
